# High‐Resolution Housing Data for Primary Prevention: What Address‐Level Records Add to National Lead‐Based‐Paint Screening

**DOI:** 10.1029/2026GH002142

**Published:** 2026-11-05

**Authors:** Eric C. Ritter

**Affiliations:** ^1^ Fluoro‐Spec Inc. East Setauket NY USA

**Keywords:** childhood lead exposure, lead‐based paint, exposure pathway, house dust, residential soil, parcel data, property records, address‐level risk, spatial resolution, modifiable areal unit problem

## Abstract

Deteriorating lead‐based paint in housing built before the 1978 residential ban remains a primary source of childhood lead exposure, reaching children through house dust and soil. Most U.S. hazard investigations follow a child's elevated blood lead test; for programs that look for hazards first, national and state maps, ours included, score housing age by census tract or county. We report a database of per‐address year‐built values for 48,634,671 homes in 41 states and D.C. (34.5 percent of U.S. housing units), assembled from 119 public assessor and parcel services and released with harvest code and record‐level provenance tooling. Harvested age distributions reproduce the independent Census estimate (Spearman 0.958 across 657 counties; tract‐level 0.74 to 0.95). We then compute, from national survey rates by construction era and at equal testing effort, what each resolution step adds to expected screening yield, the share of tested homes containing lead‐based paint. Blind canvassing yields 29.3 percent, county targeting 60.5, and ordering addresses by construction age 87.0. The tract rung reached 66.2 percent in Miami‐Dade County, Florida, where 4.6 percent of parcels predate 1940, and 84.7 in Erie County, New York, where 32.8 percent do; address ordering added 11 and 2 points. In 34,138 inspected New Jersey rentals, mostly in visual‐assessment municipalities, older homes more often had a recorded lead‐based paint hazard, also within census tracts; the yields were not tested. For proactive programs, the database ranks homes for testing. It records no occupants, and only an inspection shows whether a home holds lead paint.

## Plain Language Summary

Lead paint in old houses is one of the main ways young children in the U.S. are poisoned by lead. The paint breaks down into house dust and yard soil, which children swallow; the older a house, the more likely it holds lead paint. Most inspections by health departments follow a child's blood test showing lead. National and state lead risk maps give one number per neighborhood, an average over its houses. We built a free database of the construction years of 48,634,671 homes from property records in 41 states and Washington, D.C., and it matches Census Bureau estimates closely. Using survey rates of lead paint by building age, we estimated how many homes with lead paint each level of detail would find at equal effort: about 29 of 100 tests without regard to age, 60 in the highest‐risk counties, and 87 testing the oldest homes first. The neighborhood level in between did well where old homes are common and less well where they are a small share of the housing, where house‐level information helps most. The database ranks which homes to test first; it says nothing about who lives there, and only an inspection shows whether lead is there.

## Key Points

- A free national database of per‐address construction years for 48,634,671 homes in 41 states and D.C. is released with provenance tooling
- For proactive testing at equal effort, expected lead‐paint yield is 29.3 percent untargeted, 60.5 by county rank, and 87.0 by address age
- Address ordering beat tract targeting by 11 points where old homes were scarce (Miami‐Dade) and by 2 where they were common (Erie)

## 1 Introduction

### 1.1 The Open Question Is the Unit of Analysis

Childhood lead exposure is a settled public‐health failure with no known safe threshold (Lanphear et al., 2005; Ruckart et al., 2021). In most U.S. jurisdictions a home is examined for lead hazards after a child who lives there has an elevated blood lead test, and for that response the address is already known. Programs that look for hazards before a child is exposed have to decide which homes to examine first, and the year a home was built is the standard housing metric for that decision, applied alongside income eligibility and the presence of a young child (Sections 2.6 and 4.2). County assessor files commonly record it, though not all of them do (Section 1.4). A local program with access to its own county's file can look it up address by address, and risk models mapped at the individual tax parcel from county assessor records were published for six and then 18 North Carolina counties (Kim et al., 2008; Miranda et al., 2002).

National and state tools apply the same metric as an area share. The federal hotspots analysis (Zartarian et al., 2024), our own prior map (Ritter, 2026a), the Washington State lead exposure risk index (Washington State Department of Health, n.d.), and Pennsylvania's Environmental Health Indicators Map (Pennsylvania Department of Health, 2026) all work from census‐tract housing‐age shares from the American Community Survey, the fraction of an area's housing units built in each construction period (ACS table B25034). A federal review of U.S. lead indices and models found that they draw mainly on housing‐age and sociodemographic data, at geographic scales that differ from one index to the next (Zartarian et al., 2022). They work from area shares because the ACS reports housing age only for areas, and because the per‐address years, although public, had not been assembled into an open file of national extent (Section 1.4).

This paper takes the predictors as given and asks about the resolution at which they are applied. A census tract is a neighborhood of roughly 1,200 to 8,000 residents, and the housing‐age share that drives its score is an average over every address it contains. The hazard, by contrast, is a property of an individual home: one house was built in 1925 and very probably holds lead paint, the house next door was built in 1995 and almost certainly does not, and the average reports neither. How much that averaging costs is an empirical question, an instance of the modifiable areal unit problem (Openshaw, 1984) and, when area conclusions are read onto individuals, of the ecological fallacy (Piantadosi et al., 1988). Answering it requires the very thing the ACS cannot provide: the actual construction year of each home. The paper proposes no new risk metric. Its contributions are the per‐address build years assembled into one open national file, which we call the address surface, and the measurement of what each level of spatial aggregation discards.

### 1.2 The Exposure Pathway From Paint to Child, and Which Term of It This Paper Measures

Lead‐based paint reaches a child directly, when the child eats paint chips, and indirectly through house dust and soil (Jacobs et al., 2002). Paint that deteriorates, or that is disturbed during renovation, releases lead into settled dust. In a national survey, nearly two‐thirds of homes with deteriorated interior lead‐based paint had a dust‐lead hazard, against one‐third of homes where that paint was in good condition (Jacobs et al., 2002), and in one cohort, children whose homes underwent interior renovation had 12% higher mean blood lead by age two (Spanier et al., 2013). Soil beside a building receives lead from its exterior paint and from past gasoline emissions (Mielke & Reagan, 1998). Young children take in both media through hand‐to‐mouth contact (Lanphear & Roghmann, 1997; Levin et al., 2008), and a pooled analysis of 12 studies identified house dust as their major source of lead exposure, with soil also contributing (Lanphear et al., 1998). A model using only the approximate age of a home and whether its interior paint was peeling classified household dust lead as elevated or low with 75% accuracy (Dietrich et al., 2022).

Construction era estimates the first term of this chain, the probability that the source is present in a home (Section 2.3). It does not measure the later terms: the condition of the paint, the lead in the dust and soil, recent renovation, and whether a child under six lives in or regularly visits the home.

Resolution matters because lead in soil and dust varies from one home to the next. Lead left in soil by leaded gasoline varies across neighborhoods with historic traffic density (Mielke & Reagan, 1998; Wade et al., 2021). A building's own paint adds lead to that residue at the building itself, and soil lead is highest within a meter of the foundations of pre‐1978 homes (Wade et al., 2021). An area average of housing age spreads the source probability of an area's old homes across newer homes that mostly lack the source, and lowers it for old homes in mostly new areas. Sections 3.3 to 3.6 measure that mismatch as a loss of screening yield, the expected share of tested homes containing lead‐based paint. The address surface places the source term at the dwelling. An inspection establishes whether the paint is there, dust‐wipe and soil samples establish whether the dust and soil are contaminated, and whether a child is present has to come from occupancy information the program holds or can obtain (Section 4.2).

### 1.3 Two Failure Modes, Stated as Hypotheses

Averaging can fail in two opposite directions, and before the data are examined it is genuinely unclear how large either failure is at any given scale.

The first candidate failure is the high‐risk home hidden inside a lower‐risk area: an area dominated by new construction scores low, and the genuinely old homes inside it, each carrying the same roughly 0.87 lead‐paint probability as any pre‐1940 home anywhere, inherit a low priority they do not deserve. The second is the low‐risk home diluting a higher‐risk area: an area of mostly old housing scores high, and a program canvassing it spends visits on postban homes carrying a probability near 0.03.

Whether these failures are large depends on how housing ages are spatially arranged, and that differs by scale. Old homes cluster, because cities grow outward from historic cores, so a sufficiently fine areal unit may capture most of the clustering while a coarse one dilutes it. The results below show exactly this: the county level loses a great deal of signal, while the tract level loses much less where old housing is tightly clustered, and the address level, under a single national rate for each era, is the only unit whose expected performance does not depend on the arrangement. Every per‐home action a proactive program takes (the risk assessment, the X‐ray fluorescence [XRF] reading, the dust wipe) is spent one home at a time, so yield per visit is the correct currency for the comparison. A child's blood lead test measures exposure that has already occurred and belongs to secondary prevention, which this comparison does not address.

### 1.4 Why National Maps Work From Area Shares, and What This Paper Assembles

Prior national maps report area shares because their housing‐age source does. The ACS publishes housing age as counts of units by construction period for areas, the smallest of them the census block group, and publishes nothing for an individual address, so a model built on ACS inputs reports an area share whatever unit it chooses. Per‐address build years exist, but they live in county assessor and parcel systems, one jurisdiction at a time, in inconsistent formats, behind a patchwork of public ArcGIS feature services, Socrata and CKAN open‐data portals, bulk downloads, and in some counties paywalls or no public access at all. The sources are fragmented, and assembling them into one national surface is laborious.

Compiled national files of assessor records do exist, but none is open at the level of the individual record, in the sense that anyone may download and redistribute the records without a license or a data use agreement. Commercial providers compile and license parcel files that carry a year‐built field, and Zillow built its ZTRAX compilation partly from such a third‐party provider (Leyk & Uhl, 2018). ZTRAX held build years for more than 117 million structures and reached researchers through institutional data share agreements (Uhl et al., 2021), and the open product derived from it, HISDAC‐US, is a 250 m grid with no addresses (Leyk & Uhl, 2018). The group that built the North Carolina parcel models reports a limit in the public records themselves: its parcel models are possible only in counties whose assessor data include year built, roughly half of North Carolina's counties, so for its statewide priority list it modeled ZIP code areas, and it reports that the move lost the information available at the parcel (Callender et al., 2024).

We assembled an open file at the level of the individual record: 48,634,671 per‐address year‐built records across 41 states and the District of Columbia from 119 public sources, released in full with the harvest code and source registry under CC‐BY‐4.0 (Ritter, 2026b). With a real build year for each address, the era‐based lead‐paint probability that prior maps applied to an area average can be applied to each home, and the cost of averaging becomes directly measurable.

### 1.5 Relation to Prior Work, Including Our Own

This paper is the second in a line. The first established a free, public, tract‐level national risk map from Census housing age and poverty and validated it against measured childhood blood lead in 11 states, finding moderate agreement (Spearman rho 0.43 to 0.77) (Ritter, 2026a; hereafter paper 1). That validation is not repeated here; this paper takes the predictors as given at the area scale and asks only what resolution adds.

The federal anchor remains Zartarian et al. (2024), which scored 73,086 tracts and validated them against roughly 4.2 million Michigan and Ohio blood‐lead results, and whose authors state plainly that a tract analysis cannot identify risk at particular addresses. Risk modeling at the individual parcel is older than the national maps. Miranda et al. (2002) and Kim et al. (2008) mapped predicted lead exposure risk at the tax parcel in North Carolina counties from county assessor records, blood lead surveillance, and census data; Potash et al. (2015) predicted individual children's lead poisoning risk in Chicago; and Abernethy et al. (2018) ranked individual Flint parcels for lead service lines. Each of those models was fitted to the outcome data of one city or one state. This paper is closer in spirit to that tradition than to the national ecological maps, but it is national in extent, and it predicts paint risk from the build year itself rather than fitting per‐parcel models to local outcomes. We claim no new statistical method. The contributions are the assembled open national surface and the resolution ladder, which gives the expected difference in yield between the area shares that national and state tools use and the address‐level lookup that local programs already practice. One part of the paper's claim about address‐level records, that a home's own construction era carries information that its tract's housing‐age score does not, is checked against lead‐based paint hazards recorded at rental inspections in New Jersey (Sections 2.8 and 3.7).

## 2 Data and Methods

### 2.1 Overview

The method has six parts: a harvest of per‐address year‐built values from public property records; a data‐quality audit whose findings are reported as results rather than silently corrected; a per‐address risk score applying era‐based probabilities modified by local poverty; a validation of the harvested age distribution against the independent Census distribution; a set of equal‐effort targeting comparisons across four levels of spatial resolution; and a check, among rental homes inspected under New Jersey's lead law, of whether a home's own construction era predicts a recorded lead‐based paint hazard among homes in the same census tract (Section 2.8). All inputs are public, and the data set, source manifest, and harvest code are archived openly.

### 2.2 The Parcel and Assessor Harvest

We harvested per‐address year‐built values from 119 contributing county and state assessor and parcel sources: 116 ArcGIS feature services, two Socrata portals, and one CKAN portal. A further five configured sources returned no usable records and 11 investigated jurisdictions offered no usable publication; both sets are disclosed in the manifest. Records were normalized to a common schema: jurisdiction, parcel identifier, situs street address, integer year built, and source. A plausibility filter admits years strictly between 1500 and 2027; records with missing or unparseable years are dropped before insertion. Fourteen records in version 1.1 of the release carry years from 2 to 198 that this filter should have removed, and every analysis here excludes them. The complete manifest is Table 1.

**Table 1:** Sources

Category	Sources	Records
ArcGIS feature services	116	46,985,514
Socrata portals	2	1,199,872
CKAN portals	1	449,285
Contributing total	119	48,634,671
Configured, returned no records	5	0
Excluded, disclosed defects	2	0
Investigated, no usable publication	11	0

*Note.* Contributing sources by transport; the full per‐source manifest (jurisdiction, endpoint, record count, year‐built field, harvest date) is archived as sources_manifest.csv, with configured‐but‐empty, excluded, and no‐publication jurisdictions flagged in a contributing column.

*Deduplication and audit*. A post‐harvest audit found three classes of defect that are characteristic risks of assembling a national surface from independently maintained jurisdictional systems: two mechanisms of record duplication (exact records re‐inserted by harvester re‐runs, and the same parcels published under two different source labels) and one year‐built encoding that cannot be reliably resolved to a calendar year.

First, re‐runs of the sharded harvester had re‐inserted 4,037,444 exact‐duplicate records (identical parcel identifier, year, address, and source); 34 of 42 jurisdictions were affected, in proportions from under 0.1% to one small source at 10‐fold inflation. The duplication was detected by tuple‐level counting, removed, and independently confirmed by a consistency test: before deduplication two states reported more harvested records than the Census counts housing units in them, which is impossible, and after deduplication no state does. Because a four‐field key could in principle also collapse legitimate condominium units sharing a parcel and address, the removed records' copy‐multiplicity was examined per source: the worst‐affected sources show the signature of whole‐shard re‐insertion rather than of building sizes (99.6% of Idaho's duplicated keys occur exactly 10 times; Florida's cluster at two and three copies, and Florida assigns each condominium unit its own parcel folio, so unit collapse there would require differing identifiers the key preserves). The multiplicity distributions ship with the data set.

Second, 456,311 further records were the same parcels published under two different source labels, invisible to the exact‐duplicate pass: Manhattan appears in both the New York statewide parcel mirror and the New York City PLUTO source (40,026 records; PLUTO retained as authoritative), and Hennepin County, Minnesota appears in both the statewide source and a Metropolitan Council metro source (416,285 records, matched exactly on the assessor parcel identifier; the sources were merged so parcels unique to either survive). Candidate overlaps elsewhere were examined and rejected as coincidences of blank address fields or of identical street names in different counties; the acceptance rule for genuine duplication was same‐county overlap on both sides approaching totality.

Third, one source (San Diego County, California, 902,107 records) publishes its year‐built field as a two‐digit code that cannot be reliably resolved to a calendar year and is withheld from this version pending re‐harvest, and a smaller New York source duplicated by the statewide mirror was excluded outright.

The cleaned set contains 48,634,671 addresses across 41 states and D.C. Nine states (Arkansas, Kansas, Kentucky, Louisiana, Maine, Mississippi, New Hampshire, Rhode Island, and Vermont) have no usable machine‐readable statewide year‐built publication and are absent. Every exclusion and both deduplication passes are disclosed machine‐readably in the released data set.

*Record‐level provenance*. Every record carries a source tag, and the release includes a provenance map resolving each tag to the publishing system's live query endpoint, parcel‐identifier field, and transport. For sources holding 94.0% of records, any single record can be retrieved from the official government system by its parcel identifier through a one‐line query; the remainder resolve to their source layer or portal. This property was verified rather than assumed: on 2026‐07‐29 a sampled record from every source was queried against its live endpoint, 113 of 119 sources returned the exact record (one endpoint had been relocated by its county and is disclosed as such), and the per‐source results ship with the data set (provenance_verification.json). The release includes a dependency‐free command‐line tool (audit.py) that performs the full loop offline: given an address, it finds the matching records in the downloaded files, prints each record's official‐source link, and on request queries the government system live and reports whether the source's current value agrees with the data set. The data set is therefore auditable at the level of the individual value, without reference to the author; an area‐level average cannot be audited this way, because an average has no source record to check. Source endpoints are operated by their governments and can move; the map records them as of the June 2026 harvest, and the harvest registry allows re‐resolution.

### 2.3 The Per‐Address Risk Score

Each address receives a lead‐based‐paint probability from its construction era (Table 2). The probabilities for homes built before 1940 (0.87), from 1940 to 1959 (0.69), and from 1960 to 1977 (0.24) are the shares of homes with lead‐based paint anywhere in the building in the 1998 to 1999 National Survey of Lead and Allergens in Housing (Clickner et al., 2002, Table 4.1). The later national survey of 2018–2019 found nearly the same shares, 85, 61, and 24% (U.S. Department of Housing and Urban Development, 2021). For homes built in 1978 or later we assign 0.03. That value is not a surveyed prevalence of lead‐based paint. The surveys found lead at or above the regulatory threshold in 6.5–7% of these homes, but in the 2018 to 2019 survey 2.7% points of the 6.5 came from homes counted only because of lead in ceramic surfaces such as glazed tile (U.S. Department of Housing and Urban Development, 2021, Tables 4–2), and 3% of homes built from 1978 to 1998 had a significant lead‐based paint hazard (Jacobs et al., 2002). Table 2 lists the four probabilities.

**Table 2:** The Per‐Address Score

Construction era	Lead‐based‐paint probability
Before 1940	0.87
1940 to 1959	0.69
1960 to 1977	0.24
1978 and later	0.03

*Note.* Era probabilities: before 1940, 0.87; 1940 to 1959, 0.69; 1960 to 1977, 0.24, the shares of homes with any lead‐based paint in the 1998 to 1999 national survey (Clickner et al., 2002, Table 4.1); 1978 and later, 0.03, an assigned value (Section 2.3). ZIP code tabulation area poverty multiplier (ACS table S1701): below 10 percent, 1.0; 10 to under 20, 1.2; 20 to under 30, 1.5; 30 and above, 1.8; missing, 1.0. S(address) = min(100, 100 × P(era) × F(poverty)). All targeting comparisons rank on the era term alone.

An area model multiplies each probability by the area's share of housing in that era and sums to an average; here each address falls in exactly one era and receives that era's probability directly. These probabilities are national survey estimates applied without regional calibration. The survey that supplies them also reports prevalence by region and era, from small samples with wide confidence intervals: for homes built before 1940 it found lead‐based paint in 86, 91, and 89% of homes in the Northeast, Midwest, and South and 71% in the West, and in the South it found lower rates than the national ones for homes built from 1940 to 1959 (54%) and 1960 to 1977 (17%) (Clickner et al., 2002, Table 4.1). A single national rate for each era is applied everywhere, so the yields for Miami‐Dade, whose address rung reaches the 1940 to 1959 band, may be overstated relative to Erie, and the county‐specific yields reported below inherit that assumption (Section 4.3). The probability is then modified by a poverty factor drawn at the address's ZIP code tabulation area from ACS table S1701, a step function taking 1.0 below 10% poverty, 1.2 from 10 to under 20, 1.5 from 20 to under 30, and 1.8 at 30% or above, defaulting to 1.0 where no poverty record exists. The per‐address score is

S(address)=min(100,100×P(era)×F(poverty))

The age term is per‐address while the poverty term is areal, so within any small area the age term carries nearly all between‐home variation. All targeting comparisons in this paper rank by the age term alone, keeping them independent of the areal component. The released files carry the build year from which the age term is computed.

### 2.4 Validation Against the Census Distribution

The harvest can be checked without blood‐lead data because the Census publishes an independent year‐built distribution (table B25034, with the field‐to‐decade mapping verified against the published variable definitions). For each of the 657 counties present in both (Table 3), we compared harvested and ACS shares of pre‐1940 and pre‐1980 housing, counting homes built in 1979 or earlier as pre‐1980 on both sides. Because the paper's claims concern the address level, the same comparison was repeated at the census‐tract level inside the two demonstration counties, where per‐parcel tract assignment exists: harvested and ACS tract‐level era shares compared across every tract with at least 30 harvested parcels. County identity itself required reconstruction: the harvest's county field carries a county name, a municipality name (Massachusetts and Connecticut, resolved through a Census county‐subdivision crosswalk), or a source‐internal code (Florida, Maryland, Nebraska, New York City), and each form was resolved to a county before the comparison. One source that the version 1.1 registry labels as Washington County, Oregon, holds taxlots of Oregon City and neighboring cities in Clackamas County, and the analyses here assign it to Clackamas County. The crosswalk and the per‐county record counts are archived with the analysis code (Section 2.7). This is a fidelity check of the harvest itself. The blood‐lead validation of the underlying predictors was done at the tract scale in paper 1 and is not repeated.

**Table 3:** Coverage, Fifteen Largest Jurisdictions by Harvested Records (All 42 Are in the Archived Coverage Table)

State	Harvested records	ACS housing units	Coverage %	Counties covered
FL	7,042,799	9,915,957	71.0	65 of 67
CA	5,475,889	14,424,442	38.0	10 of 58
TX	4,822,193	11,654,971	41.4	10 of 254
NY	2,945,469	8,494,452	34.7	51 of 62
NC	2,700,541	4,739,881	57.0	49 of 100
NJ	2,630,678	3,756,340	70.0	21 of 21
MA	2,173,166	2,999,314	72.5	14 of 14
MD	2,041,593	2,531,075	80.7	24 of 24
AZ	1,688,929	3,097,768	54.5	2 of 15
OH	1,649,376	5,251,209	31.4	7 of 88
TN	1,345,098	3,050,850	44.1	86 of 95
PA	1,252,843	5,753,908	21.8	3 of 67
UT	1,156,661	1,162,654	99.5	28 of 29
MN	1,147,960	2,493,956	46.0	11 of 87
CT	1,019,847	1,531,332	66.6	9 of 9
National	48,634,671	140,943,613	34.5	657 counties

*Note.* Housing units are ACS 2018–2022 table B25001 (link in Availability Statement). Counties covered are counties with at least one harvested record.

### 2.5 The Two Demonstration Counties at Tract Resolution

Two counties were selected before the tract analysis was run, as opposite extremes of county rank and housing age: Miami‐Dade County, Florida, a newer‐built county that county‐level ranking places low, and Erie County, New York, an old‐housing county ranked in the top national decile. For each, the analysis set is a direct pull from the county's own public parcel service rather than the national bundle, so the demonstration is end‐to‐end reproducible from primary sources: 514,536 Miami‐Dade parcels with a documented build year from the Florida statewide cadastral service, and 319,925 Erie parcels from the New York State tax‐parcel centroid service. Each parcel's coordinates (published point, or polygon centroid computed client‐side) were assigned to a 2020 census tract by point‐in‐polygon test against the 2024 Census TIGER/Line tract boundaries (link in Availability Statement), with 100% assignment and zero out‐of‐county assignments in both counties, and joined to the tract risk percentiles archived with paper 1. The centroid method was cross‐validated against independent street‐address batch geocoding on the subset both could resolve: the two methods agreed on the tract for 99.83% of addresses.

### 2.6 Equal‐Effort Targeting Comparisons: The Resolution Ladder

The yield comparison holds effort fixed and varies only the information used to aim it. For a testing budget equal to 10% of a jurisdiction's addresses:

- *Blind canvass*: homes tested without regard to age. No proactive program is likely to work this way; the rung is the reference point against which targeting is measured. Expected yield is the area's mean era probability, which is exactly what a perfectly calibrated area score reports for that area.
- *County targeting*: counties ranked by their housing‐age score (the age component of the paper 1 index, which excludes its poverty term), whole counties added until they cover 10 percent of all matched addresses, every address inside them canvassed. The county that crosses the budget is included, and the three counties tied on the rounded score at the 10 percent cut are ordered by harvested record count, largest first; other orders give 89 counties and 60.4 to 60.7 percent. Because that score's values are not themselves percentiles, all threshold comparisons use the empirical percentile cutoffs of its distribution across the 3,222 scored counties, and county percentiles quoted in this paper refer to that distribution.
- *Tract targeting*: within a county, tracts ranked by the mean era probability of their own harvested parcels (age information only, matching the other rungs), filled highest first to the same 10 percent budget, everything inside a selected tract canvassed. The tract that crosses the budget is included; counting it pro rata instead changes no tract yield by more than 0.3 points. Measured in the two demonstration counties, where per‐parcel tract assignment exists. Ranking tracts instead by the paper 1 composite tract percentile, which adds poverty, yields less in both counties (58.1 vs. 66.2 percent in Miami‐Dade, 82.2 vs. 84.7 in Erie at the 10 percent budget) and is reported as a sensitivity variant rather than the primary rung.
- *Address targeting*: all addresses ranked oldest era first; expected yield is the band‐probability‐weighted mean over the selected addresses, with age treated as uniform within a partially selected band.

Every rung therefore ranks on housing age alone, so differences between rungs measure spatial resolution with the ranking information held constant. Because every rung's yield is computed from the same four era probabilities, ordering addresses by era selects the highest‐yield set of homes those probabilities allow, and no coarser rung can exceed it at the same budget. The address rung is therefore an upper bound under the model, and the ladder measures how far each area rung falls below it, which depends on how much old housing there is and how it is arranged. If the prevalence of lead‐based paint within an era differs between neighborhoods, through abatement, renovation, window replacement, or regional building practice, an area rank could carry information that a build year lacks, and observed gaps would be smaller than the expected gaps reported here. Programs in practice select homes on more than age. Federal hazard‐control grants are restricted by statute to housing occupied by, or made available to, low‐income families (Residential Lead‐Based Paint Hazard Reduction Act of 1992, 2024), and rehabilitation assisted under programs of the U.S. Department of Housing and Urban Development carries lead‐based paint requirements of its own in housing built before 1978 (Lead‐Based Paint Poisoning Prevention in Certain Residential Structures, 2026). Rules of that kind apply to a home whichever rung selects it, and they are left out of the comparison so that the rungs differ in spatial resolution and in nothing else. The ladder was not re‐measured within an income‐eligible subset of homes, because per‐address income is not public (Section 4.3).

The same construction at a 5% budget is reported alongside (Table 4); the ordering of the rungs is unchanged at both budgets.

**Table 4:** The Resolution Ladder and What Coarse Rungs Hide

Rung	National	Miami‐dade	Erie	Inspection cost per home found, at $300/at $900
Blind canvass	29.3%	31.0%	51.9%	National $1,024/$3,072; Miami‐Dade $968/$2,903; Erie $578/$1,734
County targeting, 10% budget	60.5%	Deprioritized (33rd percentile)	Selected (94th percentile)	National $496/$1,488
Tract targeting, 10% budget	Not measurable nationally	66.2%	84.7%	Miami‐Dade $453/$1,360; Erie $354/$1,063
Tract targeting, 5% budget	Not measurable nationally	67.9%	85.4%	Miami‐Dade $442/$1,325; Erie $351/$1,054
Address ordering, 10% budget	87.0%	77.2%	87.0%	National and Erie $345/$1,034; Miami‐Dade $389/$1,166
Address ordering, 5% budget	87.0%	85.4%	87.0%	National and Erie $345/$1,034; Miami‐Dade $351/$1,054

*Note.* Expected lead‐based‐paint hit rates (yields) at the stated testing budget; every rung ranks on housing age alone. The last column is the expected inspection cost per home found with lead‐based paint, the inspection price divided by the yield, rounded to the dollar, at $300 and at $900 per inspection. Those are the two ends of the range that the majority of certified lead inspectors and risk assessors in GAO's national survey charged for a lead‐based paint inspection of a single‐family home in 2019 (U.S. Government Accountability Office, 2021), not adjusted for inflation. Each cost is labeled with the geography whose yield it uses; the tract rung has no national yield, and the county rung is measured nationally only. Hidden and skippable stock at county resolution, unweighted/housing‐unit‐weighted cutoffs: pre‐1940 below the median county, 1,108,504 (15.9%/12.5%); below the 25th percentile, 440,007 (6.3%/4.3%); pre‐1960 below the median, 4,187,600 (28.5%/24.5%); post‐1978 above the 75th percentile, 2,557,290 (10.2%/10.7%).

### 2.7 Reproducibility and Provenance

All inputs are public records: assessor and parcel services for build years; the Census API for poverty, the validation distribution, and the counts of older rental housing in Section 3.7; and, for the check of Section 2.8, the New Jersey inspection dashboard and the state's inspection‐method, parcel, and municipal boundary layers. No individual blood‐lead values, records identifying a person, or access‐restricted inputs are used. The data set, source manifest, harvest code and registry, deduplication reports, provenance map, and audit tool are archived (Ritter, 2026b). Version 1.2 of the archive adds the analysis code, the county crosswalk, the per‐county record counts, the coverage table for all 42 jurisdictions, the parcel pulls and tract assignments for the two demonstration counties, and the figure code, so every number and figure outside the New Jersey check can be regenerated from the archive, the public Census tables, and the prior paper's archived scores. Version 1.3 adds the code, the analysis plan, and the aggregated outputs of the New Jersey check, and changes no record. That code re‐reads the public dashboard and keeps only submissions dated through 29 September 2026, but records on the live dashboard can still be added, corrected, or removed, so the archived outputs are the record of the run of that day and a later run may give different counts. The parcel‐level files that link the inspections to build years are not redistributed. The competing‐interest declaration is Section 6.

### 2.8 A Check Against Inspection Results in New Jersey

The yields of Sections 2.6 and 3.6 are expected values computed from national era probabilities, and under those probabilities an area rung cannot outyield the address rung (Section 2.6). One part of the claim about address‐level records can be checked against recorded outcomes: whether a home's own construction era predicts what an inspection records among homes in the same census tract, where every tract and county score takes a single value. New Jersey's rental lead law (P.L.^2021^, c.182) requires municipalities to inspect rental dwellings for lead‐based paint hazards at tenant turnover or within 2 years of the law's effective date, whichever comes first, and after that at the earlier of tenant turnover or every 3 years (N.J.S.A. 52:27D‐437.16(b)). Units built in 1978 or later are exempt, as are units certified free of lead‐based paint, units with a valid lead‐safe certification, single‐ and two‐family seasonal rentals, and units in multiple dwellings registered with the state for at least 10 years that have no outstanding lead violations (N.J.S.A. 52:27D‐437.16(c)). The New Jersey Department of Community Affairs publishes each submitted inspection on its Lead Paint Inspection Law Dashboard (link in Availability Statement) with the county, municipality, block, lot, property type, submission date, and whether lead‐based paint hazards were found. The law sets the inspection method by municipality. Where fewer than 3% of tested children aged six and under have a blood lead level of 5 μg/dL or more, inspectors may use visual assessment, which the law defines as “a visual examination for deteriorated paint or visible surface dust, debris, or residue” (N.J.S.A. 52:27D‐437.16(a)). Elsewhere they must use dust wipe sampling, in which a wipe of a representative surface is tested by a method approved by the U.S. Department of Housing and Urban Development (N.J.S.A. 52:27D‐437.16(a), (g)); below the threshold the law permits visual assessment but does not require it. Each municipality's method was taken from the layer published by the New Jersey Department of Community Affairs and New Jersey Department of Health (link in Availability Statement), in its version last edited on 20 February 2025. The dashboard's public table does not show the method used at each inspection, so that assignment is applied to every submission, including those made before the edit, and a municipality whose method changed during the period would be misassigned for part of it. The outcome of this check is therefore a lead‐based paint hazard recorded at a rental inspection. In visual‐assessment municipalities it is a finding of deteriorated paint or visible dust, and no lead is measured. In dust‐wipe municipalities it rests on a laboratory measurement of lead in house dust, although the dashboard shows only whether a hazard was found and gives neither the dust‐lead loading nor the standard against which a wipe was judged.

We used the 49,834 submissions dated 19 July 2024 to 29 September 2026, as displayed on 29 September 2026, of which 29 could not be keyed to a municipality, block, and lot. The request was limited to fields shown in the dashboard's public table and included no street address, owner, or occupant field, and no health data were read. A property was keyed by municipality, block and lot, with municipality names converted to state municipal codes through the state municipal boundary layer (New Jersey Office of GIS; link in Availability Statement) and qualifiers such as condominium unit numbers dropped, and linked by the same key to the New Jersey records of the released database (Section 2.2; Ritter, 2026b); where a key held several records, the earliest valid year (1800–2026) was used. A property was counted as positive if any of its submissions recorded a hazard, and it was scored with the era probability of Section 2.3. Each linked property was placed in a 2020 census tract (2024 TIGER/Line boundaries) by the centroid of its parcel in the statewide parcel composite (New Jersey Office of GIS) and given its tract's ACS 2018–2022 pre‐1940 share (table B25034), the tract index of paper 1, and the county housing‐age score on which the county rung ranks (Section 2.6), the last two read from paper 1's archived scores (Ritter, 2026a, 2026c). The analysis set is the linked properties with a valid year, a tract, all three area scores and a listed inspection method.

An analysis plan, kept with the code and not deposited with any registry, was written on 29 September 2026, before tracts were assigned and before any model or comparison was computed; the share of hazards by era, overall and within each inspection method, had already been seen. The New Jersey records were chosen from several public sources of property‐level lead outcomes examined in feasibility pulls on the same day. Shares by era had also been computed for some of those sources, and none of the other sources is analyzed here. The comparison that answers the question above is a conditional logistic regression of the outcome on the era probability with one stratum per cell, a cell being the homes of one census tract that lie in one municipality. Within a cell every area score is constant, and all homes are inspected under one municipality's method. Standard errors are clustered by municipality. We also report a within‐cell area under the receiver operating characteristic curve (AUC): the probability that, of two homes in the same cell, one with and one without a recorded hazard, the home with the hazard has the higher era probability, with ties counted as one half. The plan named as its primary comparisons the pooled AUC of the home's era against that of each of the two tract scores, and it also specified the pooled AUC of the county score, logistic models with area terms, and hit rates of targeting rungs among the inspected homes, all of which set homes in different municipalities against each other. Municipalities turned out to record hazards at very different rates (Section 3.7), and on seeing that we decided to report those pooled comparisons as unable to separate the measures. Several analyses were added after the results and are labeled post hoc: an AUC computed only over pairs of homes in the same municipality, for the home's era and for each tract score, including the area model of Section 2.3 applied to each tract's B25034 counts with the 1970 to 1979 count split 8 to 2 at 1978; re‐estimates of the within‐cell model in dust‐wipe municipalities, without the two municipalities that supply most of its informative pairs and with each dust‐wipe municipality left out in turn; Mantel‐Haenszel odds ratios between eras within cells; the within‐cell estimate without heaped build years, and a linear probability model with and without fixed effects for the quarter of first submission; and, for the targeting rungs, hit rates averaged over 1,000 tie‐break draws and the rates predicted by the era probabilities scaled to the observed base rate (Table S1). Confidence intervals for AUCs come from resampling municipalities with replacement (2,000 replicates). Planned sensitivity analyses repeated the within‐cell model on first submissions only, on one‐unit properties, and with the other variants listed with the code.

An AI assistant was used as a tool throughout: retrieving and processing public data, writing statistical and mapping code, searching the literature, auditing data quality, and drafting and editing text. The author directed the work, audited the pipeline, and confirmed every reported figure against an independent re‐run of the code; each reference was verified against its CrossRef or publisher record. The author takes full responsibility for the content. No AI system is an author, per ICMJE and publisher policy, because an AI cannot be accountable for the work.

### 2.9 Ethics and Human Subjects

Publicly available property, rental inspection, and Census data only; from the public New Jersey inspection records no street address, owner, occupant, or health field was read, and the parcel‐level files linking those records to build years are not redistributed; no individual human subjects, no identifiable private information, no intervention; institutional review board approval was not required. The analysis predicts risk in advance of testing and cannot determine whether lead is present in any home.

## 3 Results

### 3.1 Coverage of the Assembled Database

The database holds 48,634,671 per‐address records across 41 states and D.C. from 119 contributing sources: 34.5% of the ACS estimate of 140,943,613 U.S. housing units, and 37.2% of the housing units in the covered jurisdictions (Figure 1, Table 3). Coverage tracks what governments publish rather than any analytic choice: Florida's statewide cadastre delivers 7,042,799 records (71.0% of its ACS units), while states with county‐by‐county publication run thinner (California 38.0% with San Diego withheld, Texas 41.4, New York 34.7). No state exceeds 100% of its ACS units. A parcel is still not a housing unit: multi‐unit buildings can sit on one parcel and some parcels are non‐residential, so coverage percentages indicate completeness rather than counting dwellings.

**Figure 1:**
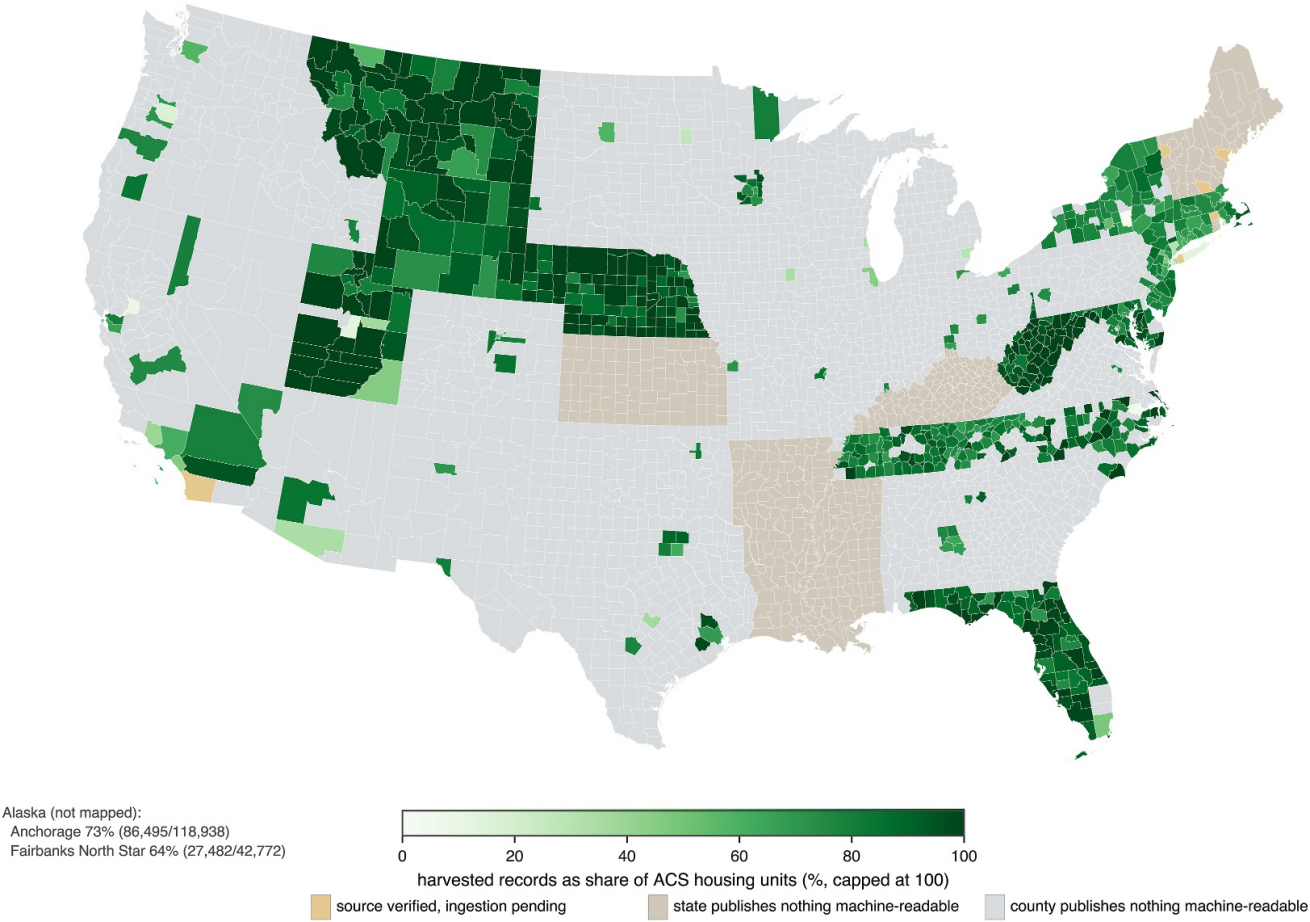
National coverage of the per‐address database. Counties of the conterminous U.S. shaded by harvested records as a share of ACS 2018–2022 housing units (sequential greens, capped at 100 percent). Non‐harvested areas are classed by records status: amber marks counties with no harvested records where a source is known but not yet ingested (Nassau County, NY, whose source is behind a paywall; Chittenden County, VT; Cumberland County, ME; Hillsborough County, NH; Providence County, RI) or whose records are withheld pending re‐harvest (San Diego County, CA); darker tan marks the rest of the nine states with no statewide machine‐readable year‐built publication (AR, KS, KY, LA, ME, MS, NH, RI, VT); light gray marks counties within otherwise‐covered states that publish none. Assessors routinely record construction years; the classes describe publication, so the map's gaps are gaps in public‐records access. Alaska (covered; Anchorage 73 percent and Fairbanks North Star 64 percent of units) is annotated rather than mapped; Hawaii, also covered, is not shown, and D.C. is mapped but too small to see at this scale. 48,634,671 records; 34.5 percent of national housing units.

### 3.2 The Harvest Reproduces the Census Age Distribution

Across the 657 counties in both sources, harvested and ACS pre‐1940 shares agree at Spearman 0.958 (Pearson 0.949), median absolute difference 3.0% points; pre‐1980 shares agree at Spearman 0.944 (Pearson 0.942) (Figure 2). Agreement holds across county size (pre‐1940 Spearman 0.928, 0.939, and 0.983 in the small, medium, and large housing‐unit terciles), so it is not carried by a few big counties.

**Figure 2:**
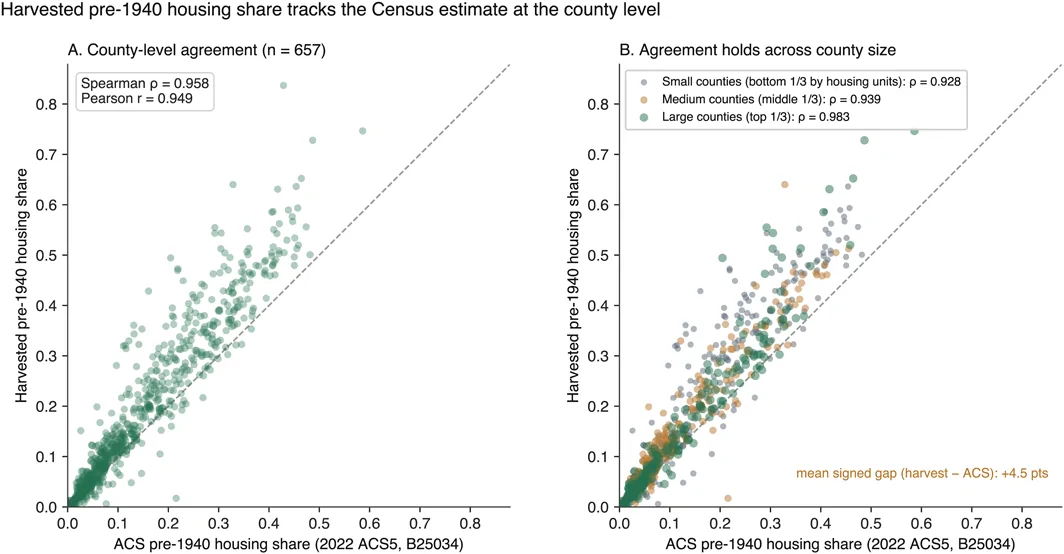
The harvest reproduces the Census age distribution. (a) Harvested versus ACS pre‐1940 housing share, one point per county (*n* = 657), 1:1 line dashed; Spearman 0.958, Pearson 0.949. (b) The same points by county housing‐unit tercile (small, medium, large), per‐tercile Spearman 0.928, 0.939, 0.983; mean signed gap +4.5 points (harvest above ACS), the expected parcel‐versus‐unit tilt.

The same check at tract resolution, inside the two demonstration counties, addresses the finer grain the paper's claims actually rest on. In Erie County (253 tracts with at least 30 harvested parcels, every one matched to an ACS tract), harvested and ACS pre‐1940 shares agree at Spearman 0.949 (Pearson 0.956); in Miami‐Dade (625 tracts, all matched), at Spearman 0.742 (Pearson 0.799) with a median absolute difference of 0.5% points, the lower rank correlation reflecting that most Miami tracts have pre‐1940 shares near zero, where ranks are unstable but absolute agreement is tight. Pre‐1980 shares agree at Spearman 0.840 (Erie) and 0.866 (Miami‐Dade). Tract‐level agreement in a condominium‐dense county and an old‐housing county is the closest available check on the per‐address years themselves rather than on their county aggregates. One systematic tilt is present and reported: the harvested pre‐1940 share runs above the ACS estimate in 78% of counties, mean signed gap +4.5 points, consistent with newer construction being disproportionately multi‐unit so that parcel weighting overweights old single‐family stock. The surface should accordingly be used to rank and locate individual old homes, which is its purpose; for estimating area composition the ACS remains the better instrument.

### 3.3 What County‐Level Aggregation Discards

All 48,634,671 addresses resolve to one of 657 counties carrying a housing‐age score. 1,108,504 pre‐1940 addresses, 15.9% of the national pre‐1940 stock, sit in counties ranked below the median; 440,007 (6.3%) below the 25th percentile. Widening to all addresses with a paint probability of at least 0.69 (built before 1960), 4,187,600 addresses, 28.5%, sit below the median. In the opposite direction, 2,557,290 post‐1978 addresses, 10.2% of the post‐ban stock, sit inside top‐quartile counties, where an undirected canvass would visit them. These shares use unweighted percentile cutoffs across the 3,222 counties; under housing‐unit‐weighted cutoffs, which shift the median toward the large counties where housing concentrates, the hidden shares are somewhat lower (12.5% of pre‐1940 stock below the weighted median, 4.3% below the weighted 25th, 24.5% of pre‐1960 stock below the weighted median) and the skippable share slightly higher (10.7%). Both are reported because neither weighting is uniquely correct; the qualitative pattern is identical. The full decile distribution (Figure 3) shows both patterns at once: pre‐1940 homes concentrate strongly upward (36.8% in the top risk decile) yet remain present in every decile, and post‐1978 homes are present everywhere including the top.

**Figure 3:**
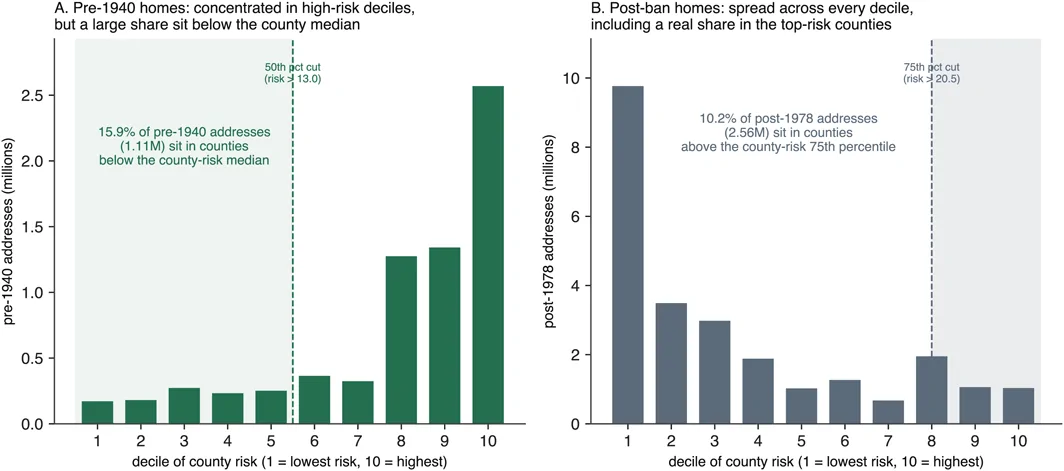
What county‐level aggregation hides. (a) Pre‐1940 addresses (millions) by decile of county risk: concentration in the top decile (36.8 percent) with 15.9 percent below the median (dashed cutoff). (b) Post‐1978 addresses by the same deciles: present in every decile, 10.2 percent above the 75th percentile cutoff inside top‐quartile counties.

### 3.4 Miami‐Dade County: The County Rung Fails, the Tract Rung Recovers Most of the Loss, and Address Ordering Adds Further Yield

Miami‐Dade holds 514,536 parcels with documented build years: 23,438 pre‐1940 (4.6%) and 230,277 post‐1978 (44.8%). County‐level ranking by housing age places it at the 33rd percentile nationally, so any allocation made by county rank deprioritizes it, and its 23,438 pre‐1940 homes, each carrying roughly 0.87 paint probability, inherit that deprioritization. This is the hidden‐homes failure at county scale, in a county holding more pre‐1940 homes than the entire harvested stock of several states.

At tract resolution the picture reverses, and this is the finding that reframed this paper: the old homes are not scattered. 88.6% of Miami‐Dade's pre‐1940 homes sit in tracts ranked above the national median of the prior paper's tract index (7 of the 23,438 sit in tracts that index does not score and are excluded from tract statistics), only 188 sit below its 25th percentile, and the modal location is the ninth risk decile (Figure 4). Miami‐Dade's old housing is spatially clustered, and a tract map captures that clustering. Consequently tract targeting inside the county yields 66.2% at the 10% budget against the county's 31.0% baseline, recovering much of what county aggregation lost, and address‐level ordering raises it further to 77.2%, an 11‐point gain over the tract rung (at a 5% budget the gain widens to 17.5 points, 85.4 vs. 67.9%; Table 4 reports both budgets). Address resolution in a mixed‐age county is not a substitute for the tract map; it is a further step of yield on top of it, and the address rung's 77.2 rather than 87.0% has a mechanical cause worth stating: the county's pre‐1940 supply (4.6% of parcels) is smaller than the 10% budget, so the oldest‐first ordering exhausts the 0.87‐probability band and continues into the 0.69 band. Address yield is bounded by local supply of old housing, never by its arrangement.

**Figure 4:**
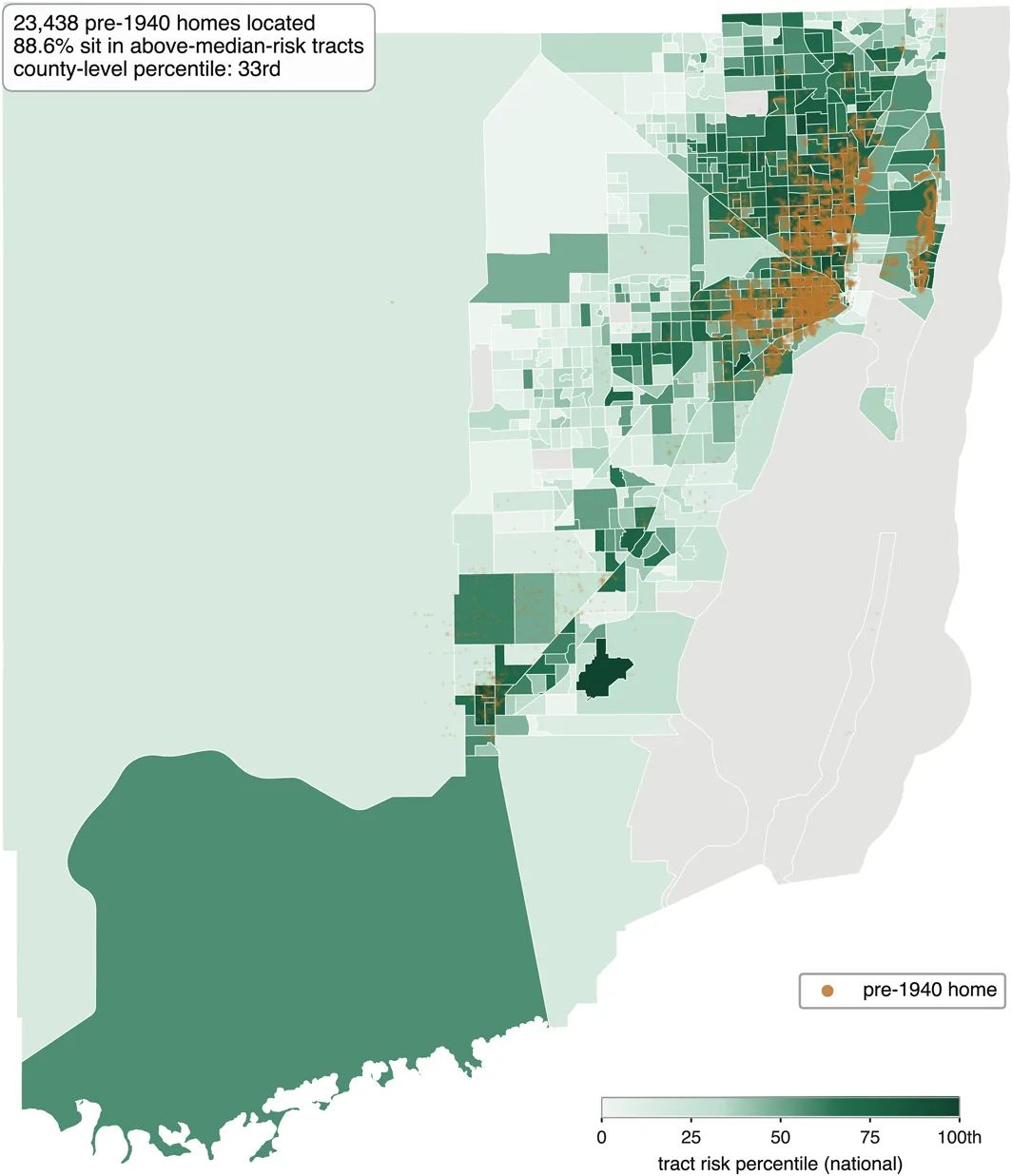
Miami‐Dade County: pre‐1940 homes cluster in above‐median tracts. Census tracts shaded by national tract‐risk percentile (greens); the county's 23,438 pre‐1940 homes as points. 88.6 percent of pre‐1940 homes sit in above‐median tracts although the county itself ranks at the 33rd percentile of the national housing‐age distribution: the county rung hides them, the tract rung finds most, and address ordering adds a further 11 points of yield.

### 3.5 Erie County: Where Housing Is Old Everywhere, the Tract Rung Nearly Suffices

Erie County holds 319,925 parcels; 319,913 carry a scored tract and all era statistics below use that set for consistency: 104,803 pre‐1940 (32.8%) and 70,462 post‐1978 (22.0%). The county ranks at the 94th percentile of the national housing‐age distribution. The dilution failure is real at county grain: roughly one blind visit in five inside this top‐ranked county lands on a post‐ban home. But the tract map again absorbs most of it: within Erie's top‐quartile tracts only 5.9% of homes are post‐1978, and within its top decile 4.6%, because the postban homes ring the county's edges while the high‐risk core is nearly free of them (Figure 5). Tract targeting yields 84.7% at the 10% budget against a 51.9% baseline, and address ordering adds two points, 87.0%. Where old housing dominates and clusters, knowing every house's age adds little to a good tract map.

**Figure 5:**
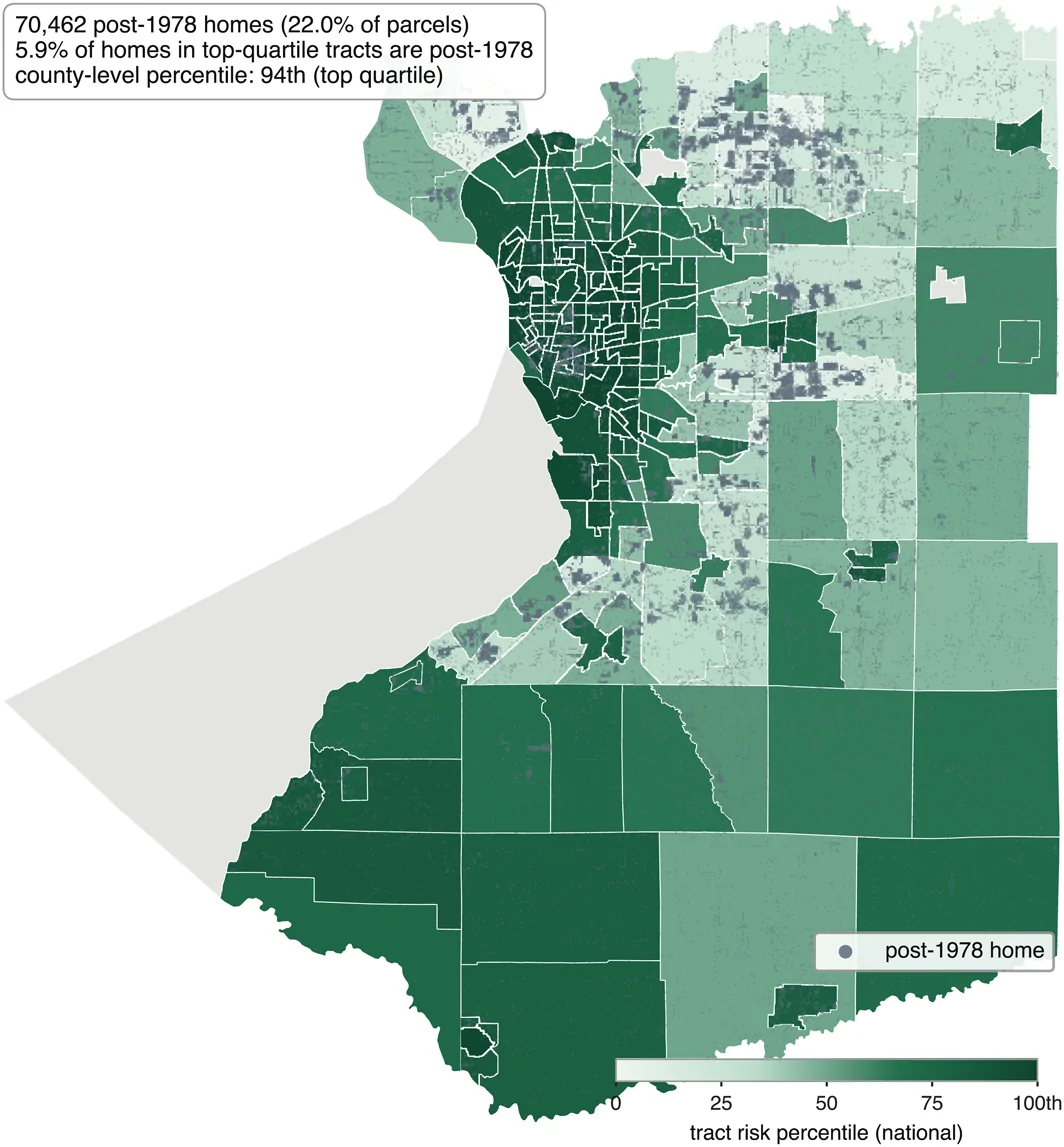
Erie County: a 94th‐percentile county whose high‐risk tracts are nearly free of postban homes. Tracts shaded as in Figure 4; the county's 70,462 scored post‐1978 homes (22.0 percent of parcels) as points, ringing the county's newer edges. Only 5.9 percent of homes inside top‐quartile tracts are post‐1978, so tract targeting already avoids most wasted visits; address ordering adds two points of yield.

### 3.6 The Resolution Ladder

Assembled nationally (Figure 6): blind canvassing yields 29.3%; county targeting at the same effort yields 60.5% (88 counties, 2.06‐fold); address‐level age ordering yields 87.0% (2.96‐fold over blind, 1.44‐fold over county). The tract rung, measured in the two demonstration counties, spans 66.2 to 84.7%, and the span itself is a finding: the tract rung's yield depends on the county's housing, how much of it is old and how tightly the old homes cluster. The address rung outyields every coarser rung in both counties and nationally, as it must under a single national rate for each era (Section 2.6); the empirical content is the size of the gap. Its behavior has a clean mechanical form: because the oldest decile of the national harvested stock is entirely pre‐1940, address‐ordered yield equals the pre‐1940 probability (87.0%) wherever local pre‐1940 supply meets the budget, as it does nationally and in Erie; where supply falls short, as in Miami‐Dade (4.6% supply against a 10% budget), the ordering exhausts the oldest band and yield reflects the next band reached (77.2%). Address‐level yield is therefore a function of how much old housing exists, independent of how it is arranged; area‐level yield depends on both.

**Figure 6:**
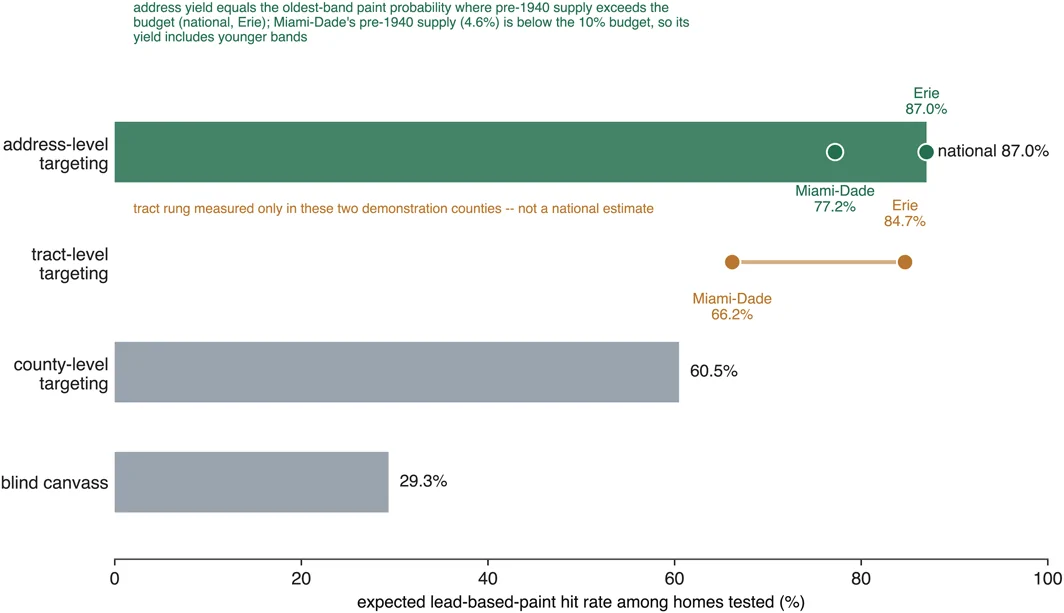
The resolution ladder. Expected lead‐based‐paint hit rate at a fixed testing budget of 10 percent of addresses: blind canvass 29.3 percent; county‐level targeting 60.5 percent; tract‐level targeting 66.2 percent (Miami‐Dade) to 84.7 percent (Erie), measured in the two demonstration counties with age‐only tract ranking; address‐level age ordering 87.0 percent nationally and in Erie, and 77.2 percent in Miami‐Dade, where pre‐1940 supply (4.6 percent of parcels) is smaller than the 10 percent budget.

Two practical corollaries follow. First, the marginal value of address resolution is largest exactly where area ranking is least informative: newer, mixed regions such as the Sun Belt, whose counties rank low while still holding tens of thousands of old homes each. Second, in old‐housing regions the address surface contributes routing efficiency rather than additional yield: it removes the residual one‐in‐17 wasted visits inside top tracts and, unlike the tract map, tells a proactive program which homes on the block are old enough to warrant a visit, before it asks which of them house a young child (Section 4.2).

### 3.7 A Check Against Inspection Results in New Jersey

Of 36,865 inspected properties, 34,251 (92.9%) linked to the released database. Linkage was lower in buildings with more units (64.7% for four or more) and in Hudson County (76.4%), and unlinked properties recorded a hazard slightly more often (12.4 against 10.0%). The dashboard covers a small and uneven share of older rental housing (post hoc): the 49,805 submissions that could be keyed equal 61.8 per 1,000 renter‐occupied units built before 1980 in the ACS 2018–2022 (table B25036), against 14.4 per 1,000 in Newark, which holds the most such units, and 183.0 in Elizabeth; the 159 municipalities with no submission hold 13.6% of those units; and among the 121 municipalities with at least 500 such units and 30 properties in the analysis set, those with more submissions per unit recorded hazards less often (Spearman −0.27). The analysis set holds 34,138 properties in 386 municipalities, and 3,424 of them (10.0%) had a recorded hazard. Of these properties, 28,633 (83.9%) are in municipalities that use visual assessment and 5,505 in municipalities that use dust wipes. The share with a recorded hazard fell with the home's era, from 11.9% (95% confidence interval [CI] 11.5 to 12.3) for homes built before 1940 to 7.6, 5.3 and 4.1% for homes built from 1940 to 1959, from 1960 to 1977, and in 1978 or later (Table 5). Because the outcome requires a hazard to be found and recorded, these rates sit far below the era probabilities for homes built before 1978 and do not estimate how common lead‐based paint is.

**Table 5:** New Jersey Rental Inspections: Recorded Lead‐Based Paint Hazards by the Home's Construction Era

A			
	All municipalities	Visual assessment	Dust wipe sampling
properties (municipalities)	34,138 (386)	28,633 (349)	5,505 (37)
with a recorded hazard	3,424 (10.0%)	2,982 (10.4%)	442 (8.0%)
built before 1940	11.9 (11.5–12.3); 22,159	12.9 (12.4–13.4); 17,537	7.9 (7.2–8.7); 4,622
built 1940 to 1959	7.6 (7.0–8.2); 7,048	7.3 (6.7–7.9); 6,397	10.3 (8.2–12.9); 651
built 1960 to 1977	5.3 (4.7–6.0); 4,714	5.4 (4.8–6.1); 4,497	3.7 (1.9–7.1); 217
built 1978 and later	4.1 (2.2–7.7); 217	4.0 (2.0–7.6); 202	6.7 (1.2–29.8); 15
within‐cell odds ratio	1.16 (1.13–1.20)	1.15 (1.12–1.19)	1.30 (1.20–1.40)
informative cells; properties; municipalities	617; 22,352; 208	536; 19,758; 181	81; 2,594; 27
within‐cell AUC of the home's era	0.547 (0.529–0.572)	0.547 (0.527–0.571)	0.553 (0.520–0.592)
pooled AUC of the home's era	0.570 (0.516–0.620)	0.586 (0.528–0.637)	0.496 (0.448–0.591)

B. All municipalities, pairs compared only within a municipality (post hoc)		
Measure	Within‐municipality AUC	home's era minus this measure
home's era	0.542 (0.526–0.578)	
tract area model (Section 2.3 probabilities on B25034 counts)	0.512 (0.499–0.536)	0.030 (0.015–0.057)
tract pre‐1940 share (B25034)	0.529 (0.508–0.540)	0.013 (−0.004 to 0.051)
tract index of paper 1	0.521 (0.492–0.537)	0.021 (−0.001 to 0.065)

*Note.* Rental properties inspected under P.L.^2021^, c.182 and entered on the state dashboard from 19 July 2024 to 29 September 2026 (link in Availability Statement), linked to the released database. Panel A gives the percent of properties with a recorded hazard (Wilson 95 percent CI; number of properties). A cell is the homes of one census tract that lie in one municipality. Odds ratios are per 0.1 of era probability, from conditional logistic regression with one stratum per cell and standard errors clustered by municipality; informative cells are those holding homes with and without a hazard. AUC intervals come from resampling municipalities (2,000 replicates). Pooled AUCs compare homes across municipalities, which record hazards at very different rates (Section 3.7). Panel B was added after the results were seen (post hoc). In visual‐assessment municipalities no lead is measured. Pooled AUCs of the area scores, all municipalities: tract pre‐1940 share 0.571 (0.434–0.700), tract index of paper 1 0.522 (0.414–0.631), county housing‐age score 0.623 (0.487–0.735). The county score is constant within a municipality and has no within‐municipality AUC.

Among homes in the same cell, a higher era probability went with a recorded hazard: conditional odds ratio 1.16 per 0.1 of era probability (CI 1.13 to 1.20), estimated from the 617 cells that held homes with and without a hazard (22,352 properties in 208 municipalities). Municipalities differ greatly in size: weighted by properties the 386 municipalities of the analysis set are equivalent to about 39 of equal size, and weighted by homes with a hazard to about 16, so the clustered intervals rest on fewer independent units than their count suggests (post hoc). Inside cells, homes built before 1940 had 1.83 times the odds of a recorded hazard of later homes (Mantel‐Haenszel, 1.63 to 2.06, interval assuming independent homes, post hoc). The within‐cell AUC was 0.547 (0.529–0.572), so the home's era separates homes with and without a recorded hazard only modestly. In the municipalities that use dust wipes, the one part of the check in which lead is measured, the odds ratio was 1.30 (1.20–1.40; 81 cells, 27 municipalities). Two of those municipalities, Irvington and Orange, supply most of the informative pairs; without them the estimate was 1.31 (1.12–1.54), and leaving out any one municipality gave 1.28 to 1.32 (post hoc). In visual‐assessment municipalities the odds ratio was 1.15 (1.12–1.19), and there the check cannot separate lead content from paint condition, since the condition of paint can vary with a building's age whether or not the paint contains lead. The within‐cell estimate was 1.16 (1.12–1.19) on each property's first submission and 1.17 (1.13–1.21) in one‐unit properties. Assessor years in the analysis set are heaped (post hoc): 41.2% end in zero, 10.3% are exactly 1900, and 947 homes carry 1940, an era boundary, against 51 to 229 in each other year from 1935 to 1944. With the heaped years 1900, 1910, 1920, 1925, 1930, 1940, 1950 and 1960 removed, the within‐cell estimate was 1.15 (1.11–1.19), and a linear probability model gave 0.91% points per 0.1 with and without fixed effects for the quarter of first submission (both post hoc). Compared only within municipalities (post hoc), the home's era had an AUC of 0.542 (0.526–0.578), against 0.512 (0.499–0.536) for the area model of Section 2.3 applied to tract B25034 counts, a difference of 0.030 (0.015–0.057). Against the tract pre‐1940 share (0.529) and the tract index of paper 1 (0.521), the differences were 0.013 (−0.004 to 0.051) and 0.021 (−0.001 to 0.065). Of the three differences only the one against the lowest‐scoring tract measure excludes zero.

Comparisons across municipalities mostly reflect how each municipality records hazards. Of the 162 municipalities with at least 30 properties, 41 recorded a hazard at fewer than 1% of them, and these hold 30.9% of the analysis set, although 63.9% of their homes were built before 1940 against 67.0% in the other 121 (Hamilton Township in Mercer County recorded none among 1,718 properties, Garfield 10 among 1,201); Montclair recorded 81.8%. By quarter of submission, the share of submissions recording a hazard ranged from 1.5% in the third quarter of 2024 to 19.5% in the second quarter of 2026. The planned pooled comparisons could not separate the measures. Pooled AUCs were 0.570 (0.516–0.620) for the home's era, 0.571 (0.434–0.700) for the tract pre‐1940 share, 0.522 (0.414–0.631) for the tract index and 0.623 (0.487–0.735) for the county score. The planned primary differences, the AUC of the home's era minus that of the tract pre‐1940 share and minus that of the tract index, were 0.000 (−0.090 to 0.091) and 0.048 (−0.018 to 0.113). Differences in how municipalities record hazards also hide the pooled era gradient among dust‐wipe municipalities (AUC 0.496): 66.4% of that group sits in three municipalities with old housing that recorded almost no hazards, and Trenton alone recorded 6 among 2,119 properties, 95% of them built before 1940. The planned comparison of targeting rungs among the inspected homes had no power in either direction. Only 217 of the 34,138 homes carry an assessor year of 1978 or later (the law exempts units built in those years), and the era probabilities, scaled to the observed base rate (post hoc), put the address rung less than a point above the tract rungs at a 10% budget (11.8 against 11.3 and 11.4%), while the bootstrap intervals on the area rungs were 20–39 points wide across the 10 and 20% budgets. In the planned single draw the hit rates at the 10% budget were 10.9% for the address rung, 15.9 for the tract pre‐1940 share, 10.6 for the tract index of paper 1, 8.7 for the county rung and 9.6 at random; averaged over 1,000 tie‐break draws (post hoc) they were 11.9, 15.9, 10.6, 8.9 and 10.0 (Table S1). The era gradient that can be tested is flatter than the probabilities: the hazard rate for homes built before 1940 was 2.2 times that for homes built from 1960 to 1977, where the probabilities give 3.6, and the contrast between homes built from 1960 to 1977 and later homes could not be estimated with useful precision (Mantel‐Haenszel odds ratio within cells 1.21, 0.42 to 3.49, interval assuming independent homes, post hoc). Errors in assessor years, and hazards recorded where deteriorated paint holds no lead, can flatten this gradient, and these data cannot separate their effect from a flatter gradient of lead‐based paint itself, which would make the observed gaps between rungs smaller than the expected gaps (Section 2.6). The check supports one part of the claim about address‐level records, that a home's own era carries information about recorded hazards among homes that share a tract. It does not test the expected yields or the ordering of the rungs.

## 4 Discussion

### 4.1 What This Paper Expected, and What the Data Said

The working hypothesis when this analysis began was that area maps hide old homes inside their polygons at every scale, and the first draft of this paper was framed accordingly. The tract‐resolution results refuted the strong version of that claim: in the very county chosen to demonstrate the hidden‐homes failure, 89% of the pre‐1940 stock sits in above‐median tracts, because old housing sits in clusters that tract maps capture. What survived measurement is a more useful conditional claim: aggregation costs yield in proportion to how finely old and new housing are mixed within the unit, at least across the two counties measured: the county rung loses a great deal everywhere, the tract rung loses little where history concentrated the old stock and more where it did not, and the address rung alone is unaffected by arrangement, bounded only by how much old housing exists relative to the budget. We report the reversal explicitly because the corrected claim is the paper's actual contribution, and because the data set that made the correction possible is released with it.

### 4.2 What Address Resolution Buys, and What It Cannot Do

Proactive targeting remains a minority of lead‐program activity; most investigations still begin with an elevated blood lead result, and for that response the address is already known. The ladder describes the choice facing programs that look for hazards first, such as grant‐funded hazard‐control programs deciding where to enroll homes or home‐visiting programs deciding whom to counsel. The county rung describes a funder or a state program choosing among counties; a local program works inside one county and faces the tract and address rungs, which Table 4 reports for the two demonstration counties. For such a program the ladder converts directly into cost: at 87.0 versus 60.5% yield, a county‐targeted program needs about 1.4 times as many visits as an address‐targeted program to find the same number of homes with lead‐based paint, and a blind program about three times as many.

The same comparison can be stated in dollars from published inspection prices. In the national survey of certified lead inspectors and risk assessors by the U.S. Government Accountability Office (GAO), the majority charged $300 to $900 for a lead‐based paint inspection of a single‐family home in 2019, the lower figure being the survey's lowest response category, $300 or less (U.S. Government Accountability Office, 2021). The economic analysis of the 2024 dust‐lead rule by the U.S. Environmental Protection Agency gives approximately $300 to $400 for an average lead‐based paint inspection, in a footnote that bases the figure on a 2022 internet search of inspection rates (U.S. Environmental Protection Agency, 2024). The dollar figures below use the two ends of the GAO range as reported for 2019, without adjustment for inflation. The expected cost of finding one home with lead‐based paint is the inspection price divided by the yield. At $300 per inspection that cost is $345 under address ordering, $496 under county targeting, and $1,024 for a blind canvass; at $900 it is $1,034, $1,488, and $3,072 (Table 4). The ratio between rungs is the same at any price, provided an inspection costs the same at every rung. A program that orders a combined inspection and risk assessment, which the same survey priced at $500 to $1,000, pays proportionally more at every rung (U.S. Government Accountability Office, 2021). At the yields of the 10% budget, 1,000 address‐ordered inspections are expected to find 870 homes with lead‐based paint. A county‐targeted program needs 1,438 inspections to find as many, and the 438 additional inspections cost about $131,000 to $394,000 at those prices. Inside one county the gap is smaller. At $300 per inspection the tract rung costs $453 per home found in Miami‐Dade against $389 for address ordering, and $354 against $345 in Erie, where old housing is concentrated. These figures price the inspection only and are stated per home found. They apply one price to every home at every rung, although the professionals GAO surveyed reported that driving distance, type of housing, and the number of rooms and surfaces affect what they charge (U.S. Government Accountability Office, 2021); homes chosen by address ordering are more scattered than the homes of a canvassed tract or county, and that difference in travel is not priced here. The yields are expected values from the era probabilities of Section 2.3, not observed inspection results, and the hazard rates recorded at the New Jersey inspections of Section 3.7 measure a different outcome and do not estimate them. The comparison also assumes that inspections are ordered from the area ranking alone. A program that checks each home's build year before ordering an inspection, as a hazard‐control grantee working in pre‐1978 housing does, is already using address‐level information, which is what the surface supplies across county lines; for such a program the coarse rungs describe where outreach is aimed. The paper has no count of the proactive inspections performed in the United States, so no national total is offered.

The surface still does not measure lead in any home. A build year is a prior rather than a test: roughly 13 in 100 pre‐1940 homes hold no lead paint, and 6 or 7 in 100 postban homes have a lead reading at the regulatory threshold, many of them on ceramic tile rather than paint (U.S. Department of Housing and Urban Development, 2021). Only an on‐site evaluation by a certified inspector or risk assessor establishes what a specific home holds: a lead‐based paint inspection, by XRF or paint‐chip analysis, for the paint, and a risk assessment with dust‐wipe and soil sampling for the hazards. The surface ranks where that test should go first, the same line between screening and diagnosis that paper 1 drew, moved from the neighborhood to the door.

The surface also records nothing about who lives in a home, and childhood exposure requires a child. The age ranking is meant to be combined with occupancy information that a program already holds or can obtain under a data‐sharing agreement, such as birth records and enrollment in Medicaid or the Special Supplemental Nutrition Program for Women, Infants, and Children (WIC), or the household information it collects at intake, so that the first visits go to old homes where a young child lives or is expected. Federal hazard‐control grants already carry a child‐occupancy condition: in owner‐occupied housing, at least 90% of assisted units must be occupied by a child under six or be units where such a child spends a significant amount of time visiting, and in assisted rental housing the landlord must give priority to families with a child under six for at least three years after the abatement work (Residential Lead‐Based Paint Hazard Reduction Act of 1992, 2024). Whether ordering homes by build year moves a program toward or away from young children cannot be measured from the harvest, which holds no occupancy record. The nearest available check is at the tract scale and uses a different ranking: in the tract index of paper 1, which combines housing age and poverty, predicted risk and the under‐five share of the population are nearly uncorrelated (Spearman 0.065 across 83,373 tracts; Ritter, 2026a), so that ranking neither concentrates on tracts with young children nor avoids them. Lead‐based paint also remains in a home when its occupants change, so an old home with no child this year still holds the paint and may house a young child in a later year.

The resolution argument carries to one part of the soil pathway, the soil beside the building. Residential soil lead is common where it has been sampled: in a citizen‐science database of 15,595 residential soil samples, nearly one quarter were at or above 200 ppm, EPA's screening level for residential soil (Filippelli et al., 2024). In a household‐scale study of 462 homes in Greensboro, North Carolina, soil lead at the drip line rose about 6% for each decade of house age after adjustment for other factors, an association not seen in yard or street‐side soil (Obeng‐Gyasi et al., 2021), and in Indianapolis the mean lead concentration of soil sampled at drip lines was more than twice that of yard soil (Filippelli et al., 2018). Soil at a foundation also collects airborne lead that settled on the building and washed down while leaded gasoline was in use (Mielke & Reagan, 1998), and the Indianapolis authors did not rule that source out, so drip‐line enrichment cannot be assigned to paint alone. To the extent that drip‐line lead comes from a building's own paint, an area average of housing age misplaces it along with the paint, although this paper measured construction years and no soil. Lead deposited on soil from leaded gasoline reflects historic traffic density (Mielke & Reagan, 1998), and a build year does not measure it.

### 4.3 Limitations

*Coverage is incomplete and uneven*. 41 states and D.C., 34.5% of national housing units; nine states publish no usable data and within covered states counties are missing. The address advantage is thinnest where open property data is thinnest; this data‐access gap parallels the surveillance gap that motivated paper 1.

*The national ladder's middle rung is demonstrated rather than nationally measured*. The harvest carries jurisdiction but not coordinates for every source, so tract assignment exists only for the two demonstration counties, chosen as opposite extremes of housing arrangement. The obvious next version of this data set is a re‐harvest that retains geometry, allowing the national tract‐rung distribution between those extremes to be measured.

*Parcels are not housing units*. The +4.5 point parcel‐versus‐unit tilt in pre‐1940 shares (Section 3.2) means area composition should come from the ACS; counts and locations of individual old homes, which is what this paper uses, are unaffected in kind. One proposed mechanism (newer construction being disproportionately multi‐unit) is plausible but untested here.

*Data quality in source systems is a live risk*. The audit found duplication (8.5% of the raw harvest across two mechanisms) and one un‐decodable year encoding, all caught by cross‐checks rather than by any source's own validation, all disclosed in the release. The deduplication key cannot distinguish a re‐inserted record from a hypothetical condominium unit published identically to its neighbor; the multiplicity analysis of Section 2.2 supports re‐insertion as the mechanism in the worst‐affected sources, but a residual risk of collapsing legitimate identical records remains wherever a source publishes units without distinguishing identifiers. Comparable efforts should assume such defects and design the checks first; a unique‐key constraint at ingestion would have prevented the largest class outright.

*The reported shares and yields depend on the placement of the county percentile cutoffs and on an era‐to‐probability mapping applied without regional calibration*. The hidden‐homes shares of Section 3.3 depend on where the percentile cutoffs fall across counties; unweighted and housing‐unit‐weighted versions are both reported, and they differ by up to 4.0% points without changing the pattern. The era‐to‐probability mapping is a national survey estimate applied without regional calibration (Section 2.3), so county‐specific yields inherit a uniformity assumption that cannot be tested from public data; the flatter era gradient of recorded hazards in New Jersey (Section 3.7) concerns a different outcome and does not test the mapping. And the harvest's fidelity is validated at county scale nationally but at tract scale only inside the two demonstration counties; per‐address years elsewhere rest on the sources themselves plus the record‐level audit trail.

*The poverty modifier is areal*. Per‐address income is not public; within small areas the factor is nearly constant, so it does not disturb the within‐area orderings used here, but the composite score is not fully individual.

*The demonstration counties are two, by design*. They bound the tract rung rather than estimate its national distribution; quantifying how yield loss depends on the mixing of old and new housing (e.g., yield gap as a function of a within‐tract age‐entropy measure) is future work the released data supports.

*Occupancy and paint condition are not recorded*. The surface holds a build year and nothing else about a home. Whether a child lives there, what condition the paint is in, and whether the home has been renovated decide whether lead‐based paint becomes an exposure (Section 1.2), and a program has to supply them from its own records or from the visit.

*Lead from sources other than the home's own paint is outside scope*. Service lines, soil lead deposited by gasoline and industry, imported goods, and take‐home occupational lead are real pathways that the lead‐based‐paint probabilities used here do not represent.

*The inspection check is narrow*. It covers rental dwellings inspected and entered on the New Jersey dashboard from July 2024 to September 2026 and linked to the released records, most of them in municipalities that assess visually for deteriorated paint and that record hazards at very different rates, so it shows that a home's era predicts a recorded hazard among homes in the same tract and tests neither the expected yields nor the ordering of the rungs.

### 4.4 Ethics

Only public property, inspection, and Census data are used: per‐address build years are public assessor records, properties of homes rather than people; the check of Section 2.8 uses the public New Jersey inspection records, from which no street address, owner, occupant, or health field was read; and no blood‐lead, health, or identifiable resident data enter the analysis. The parcel‐level files that link those inspections to build years are not redistributed. A provider can apply the surface to a local address list without transmitting resident information. The analysis allocates by build year and area poverty and does not use race or any protected characteristic. Paper 1 reported that its area model's residual was most strongly predicted by the share of Black population, meaning area maps under‐rank historically disinvested neighborhoods relative to measured burden; nothing in the present surface corrects that pattern, and race should not be an input to either model.

### 4.5 Applications

Local programs with access to their own county assessor file already look up individual build years, and the resolution ladder gives the expected screening yield that an area share loses relative to that practice. The data set puts records that governments already publish into one format with one lookup, for programs working across county or state lines and for offices in covered jurisdictions without in‐house parcel access; it adds nothing where a county publishes no build years (Section 4.3). A health department or hazard‐control grantee planning proactive outreach, or a home‐visiting or WIC program deciding whom to counsel first, can look up a specific address rather than its neighborhood by searching the released files with the bundled audit tool, or by joining them to a local address list, offline, free and without registration. A program that starts from a list of young children can attach a build year to each child's address in the same way. For each match the tool prints the link to the official source, which for sources holding 94.0% of records is that parcel's own record in the publishing government system, so an individual result can be checked against its source. The regions that gain most are the ones where current maps are least informative: newer, mixed counties whose area rank is low while their absolute old‐housing stock is large. Miami‐Dade alone holds 23,438 locatable pre‐1940 homes inside a county ranked at the 33rd percentile.

## 5 Conclusion

The hazard of lead‐based paint belongs to individual homes, while national and state lead risk maps report housing age as averages over areas. Using an openly released national database of construction years for 48,634,671 homes, validated against the independent Census distribution at rank correlation 0.96, we computed the expected yield of each level of aggregation at equal testing effort, using national survey rates of lead‐based paint by construction era: 29.3% with no geography, 60.5% with county targeting, 66.2 to 84.7% with tract targeting depending on the county, and up to 87.0% with address‐level ordering. Because each yield is the mean era probability of the homes selected, ordering by each home's own era cannot yield less than an area ranking; what the data determine is the size of the gap, which the New Jersey inspection records could not test (Section 3.7). Area maps fail worst at coarse grain and in mixed places; under a single national rate for each era, the address surface is the only rung whose expected performance does not depend on how history arranged the housing, only on how much old housing exists, and for 41 states and D.C. it is now assembled in an open file. The surface ranks homes for testing and does not diagnose them. It holds no information on who lives in a home, and a program has to add where young children live from its own records. Whether a hazard is actually present can be determined only by testing the home, and the purpose of the surface is to raise the expected yield of that testing.

## Conflict of Interest

The author is the founder of Fluoro‐Spec Inc., which manufactures and sells a consumer lead‐screening test based on methylammonium bromide, a reagent that reacts with lead in paint to produce visible fluorescence, and owns Spirochaete Research Labs, LLC, which has interests in lead‐detection technology. The author is a named inventor on pending U.S. patent applications on the measurement of lead. The conflict is direct and named plainly: this paper argues that lead screening is under‐targeted and that high‐risk addresses warrant in‐home confirmation, a policy from which the author's product could benefit commercially. The mitigations are that the method applies established era probabilities from federal surveys and follows the prior literature's targeting logic; that the paper does not evaluate, recommend, or price any consumer test, and the confirmation step it names and prices is an on‐site evaluation by a certified inspector or risk assessor; and that the entire data set, the harvest code, and the analysis code, including that of the New Jersey check, are archived openly, so every result can be rebuilt, or for the New Jersey check rerun against the public inspection records (Section 2.7), and every record checked against public records without relying on the author. For access to the data the manuscript points readers to the public archive and its offline audit tool, and to no website operated by the author's company. The strongest mitigation is record‐level auditability (Section 2.2): 94.0% of the data set's individual values resolve to a live query of the government system that published them, confirmed by sampled live queries (one record from every source, with per‐source results shipped in the release), so the data underlying every claim can be checked against its official source, record by record. Rather than assert that the commercial interest did not influence the work, which cannot be proven, the mitigation offered is verifiability of the underlying records, together with the robustness checks of Section 2.6 on the analytical choices with the most leverage on the results.

## Supporting information

Table S1

## Data Availability

All inputs are public. Per‐address build years were harvested from public county and state assessor and parcel services; housing units (B25001), poverty (S1701), the validation distribution (B25034), and the renter‐occupied units by year built used in the New Jersey check (B25036) come from the American Community Survey 2018–2022 5‐year estimates, read through the public Census API (data set https://api.census.gov/data/2022/acs/acs5 and, for S1701, https://api.census.gov/data/2022/acs/acs5/subject). The four tables are at https://data.census.gov/table/ACSDT5Y2022.B25001, https://data.census.gov/table/ACSST5Y2022.S1701, https://data.census.gov/table/ACSDT5Y2022.B25034, and https://data.census.gov/table/ACSDT5Y2022.B25036. The complete data set (48,634,671 records, 42 per‐jurisdiction files), source manifest, per‐state counts, disclosed exclusions and deduplication report, harvest code, source registry, provenance map, live‐verification results, and audit tool are archived under CC‐BY‐4.0 at Zenodo (Ritter, 2026b): concept doi:10.5281/zenodo.21685186, which resolves to the current version (version 1.3, doi:10.5281/zenodo.23049891). The records are those of version 1.1, the deduplicated release, and the version notes document version 1.0 and its differences. Version 1.2 adds the analysis and figure code, the county crosswalk, the per‐county record counts, the coverage table, and the parcel pulls for the two demonstration counties, and changes no record. Version 1.3 adds the code, the analysis plan, and the aggregated outputs of the New Jersey check of Section 2.8, and changes no record. Derived area scores and code from the prior paper (Ritter, 2026c): doi:10.5281/zenodo.20596316. The New Jersey inspection records are from the Lead Paint Inspection Law Dashboard of the New Jersey Department of Community Affairs, a Power BI report updated continuously and read as displayed on 29 September 2026 (https://datahub.dca.nj.gov/documents/njdca::lead‐paint‐inspection‐law‐dashboard). The inspection method of each municipality is from the Lead Based Paint Inspection Methods in NJ Municipalities layer of the New Jersey Department of Community Affairs and New Jersey Department of Health, version last edited 20 February 2025 (https://services.arcgis.com/Aur8tCo478N3VovT/arcgis/rest/services/Lead_Based_Paint_Inspection_Methods_in_NJ_Municipalities/FeatureServer/6). Parcel centroids are from the New Jersey Office of GIS Parcels and MOD‐IV Composite layer (https://services2.arcgis.com/XVOqAjTOJ5P6ngMu/arcgis/rest/services/Parcels_Composite_NJ_WM/FeatureServer/0), and municipal codes from its Municipal Boundaries of NJ layer (https://services2.arcgis.com/XVOqAjTOJ5P6ngMu/arcgis/rest/services/NJ_Municipalities_3857/FeatureServer/0), both read 29 September 2026. Census tract boundaries are the 2024 TIGER/Line census tract files of the U.S. Census Bureau, state files for Florida, New Jersey, and New York (https://www2.census.gov/geo/tiger/TIGER2024/TRACT/). The archived code retrieves the dashboard's public table with the same query the dashboard sends and prints a checksum for comparison with the extract used here (Ritter, 2026b). The code for the check and its aggregated outputs are archived in version 1.3 (Ritter, 2026b), and the parcel‐level files that link inspections to build years are not redistributed. Every reported result outside the New Jersey check, including all of Sections 3.1 to 3.6, rebuilds from the archive together with the public Census tables and the prior paper's archived scores. The code of the New Jersey check re‐reads the public dashboard and keeps only submissions dated through 29 September 2026, but records on the live dashboard can still be added, corrected, or removed, so the archived aggregated outputs are the record of the run of that day and a later run may give different counts.
