# Supplementary material for: High‐Resolution Housing Data for Primary Prevention: What Address‐Level Records Add to National Lead‐Based‐Paint Screening
Source: Geohealth. 2026 Nov 5;10(11):e2026GH002142. doi: 10.1029/2026GH002142 (PMC13736787; doi:10.1029/2026GH002142)
Supplement: Supplementary file 1 [file GH2-10-e2026GH002142-s001.pdf]

GeoHealth

Supporting Information for

High-Resolution Housing Data for Primary Prevention: What Address-Level Records Add to National Lead-Based-Paint Screening

Eric C. Ritter

Fluoro-Spec Inc., East Setauket, New York, USA

Contents of this file

Table S1

Introduction

This file holds Table S1, the planned comparison of targeting rungs among the New Jersey rental properties analyzed in Sections 2.8 and 3.7 of the main text. As in the main text, paper 1 is the companion paper that mapped lead risk nationally by census tract (Ritter, 2026a). Section 3.7 summarizes the comparison briefly because it has no power in either direction. The code, the analysis plan, and the aggregated outputs from which every value in Table S1 is taken are archived in version 1.3 of the dataset record (Ritter, 2026b; doi:10.5281/zenodo.23049891).

Table S1. Planned comparison of targeting rungs among the inspected New Jersey rental properties. The analysis set of Section 2.8 holds 34,138 properties, of which 3,424 (10.0 percent) had a recorded lead-based paint hazard. Each rung orders the properties by one measure and selects the first 10 or 20 percent of them (k = 3,414 or 6,828 properties; both budgets were fixed in the analysis plan, which used 20 percent where Table 4 of the main text uses 5 percent); the hit rate is the percent of selected properties with a recorded hazard. Random order uses no information. The county rung ranks counties by the housing-age score used for the county rung of Section 2.6. The two tract rungs rank all of the state's census tracts by the tract's ACS 2018-2022 pre-1940 share (table B25034) and by the tract index of paper 1. They differ from the tract rung of Section 2.6, which ranks the tracts of one county by the mean era probability of their harvested parcels, so that rung is not tested here. The address rung orders homes by construction era, oldest first. Counties and tracts are taken whole in rank order, the county or tract that crosses the budget is filled by a random draw of its homes so that every rung selects exactly k properties, and ties in rank are broken at random. The single draw is the one seeded tie-break draw fixed in the analysis plan, and its 95 percent interval comes from resampling municipalities with replacement (2,000 replicates). The expectation is the mean hit rate over 1,000 tie-break draws. The expectation and the prediction described below were added after the results were seen (post hoc). The analysis set holds more homes

built before 1940 (22,159) than either budget selects, so at both budgets the address rung is a random draw of pre-1940 homes and its expectation is the pre-1940 hazard rate, 11.9 percent. The prediction is the hit rate that the era probabilities of Section 2.3 give for each selection once scaled to the observed base rate: the selection's mean era probability multiplied by the ratio of the observed hazard rate to the mean era probability of the analysis set. The last column gives the single-draw hit rate of the address rung minus that of each other rung, with its municipality bootstrap interval. The comparison has no power in either direction. Only 217 of the 34,138 homes carry an assessor year of 1978 or later, so at either budget the scaled probabilities put the address rung less than one point above the tract rungs (11.8 against 11.3 and 11.4 percent), while the bootstrap intervals of the three area rungs are 20 to 39 points wide. The observed rates of the area rungs also depart from the rates obtained by applying the observed hazard rate of each era to the era mix of their own selections (11.4 and 10.4 percent), by 4.5 points for the tract pre-1940 share rung at the 10 percent budget and by 7.8 points for the county rung at the 20 percent budget, because municipalities record hazards at very different rates (Section 3.7). The observed differences between rungs therefore neither support nor contradict the expected yields of Section 3.6. All values are percentages.

budget	rung	single draw (95% interval)	expectation over tie-breaks	predicted from scaled era probabilities	address rung minus this rung (95% interval)
10%	random order	9.6 (6.4 to 14.8)	10.0	10.0	1.3 (0.0 to 4.4)
10%	county housing-age score	8.7 (2.9 to 33.7)	8.9	10.2	2.3 (-19.1 to 12.1)
10%	tract pre-1940 share	15.9 (2.7 to 41.6)	15.9	11.3	-5.0 (-24.2 to 6.3)
10%	tract index of paper 1	10.6 (1.0 to 30.8)	10.6	11.4	0.4 (-13.6 to 11.3)
10%	address (home era, oldest first)	10.9 (7.1 to 18.5)	11.9	11.8	
20%	random order	9.5 (6.4 to 14.6)	10.0	10.0	1.8 (0.2 to 4.3)
20%	county housing-age score	18.2 (5.7 to 33.1)	18.2	10.5	-6.9 (-17.7 to 5.1)
20%	tract pre-1940 share	14.0 (4.5 to 29.9)	14.0	11.3	-2.7 (-12.1 to 4.4)
20%	tract index of paper 1	9.6 (2.6 to 22.6)	9.6	11.4	1.7 (-4.8 to 6.4)
20%	address (home era, oldest first)	11.3 (7.1 to 18.4)	11.9	11.8	
